# Long non-coding RNA PVT1 activates hepatic stellate cells through competitively binding microRNA-152

**DOI:** 10.18632/oncotarget.11709

**Published:** 2016-08-30

**Authors:** Jianjian Zheng, Fujun Yu, Peihong Dong, Limei Wu, Yuan Zhang, Yanwei Hu, Lei Zheng

**Affiliations:** ^1^ Department of Laboratory Medicine, Nanfang Hospital, Southern Medical University, Guangzhou, Guangdong, P.R. China; ^2^ Key Laboratory of Surgery, the First Affiliated Hospital of Wenzhou Medical University, Wenzhou, Zhejiang, P.R. China; ^3^ Department of Infectious Diseases, the First Affiliated Hospital of Wenzhou Medical University, Wenzhou, Zhejiang, P.R. China

**Keywords:** plasmacytoma variant translocation 1 (PVT1), microRNA-152, DNA methylation, patched1 (PTCH1), Pathology Section

## Abstract

Epithelial-mesenchymal transition (EMT) process is considered as a key event in the activation of hepatic stellate cells (HSCs). Hedgehog (Hh) pathway is known to be required for EMT process. Long non-coding RNAs (lncRNAs) have been reported to be involved in a wide range of biological processes. Plasmacytoma variant translocation 1 (PVT1), a novel lncRNA, is often up-regulated in various human cancers. However, the role of PVT1 in liver fibrosis remains undefined. In this study, PVT1 was increased in fibrotic liver tissues and activated HSCs. Depletion of PVT1 attenuated collagen deposits *in vivo*. *In vitro*, PVT1 down-regulation inhibited HSC activation including the reduction of HSC proliferation, α-SMA and type I collagen. Further studies showed that PVT1 knockdown suppressed HSC activation was through inhibiting EMT process and Hh pathway. Patched1 (PTCH1), a negative regulator factor of Hh pathway, was enhanced by PVT1 knockdown. PTCH1 demethylation caused by miR-152 was responsible for the effects of PVT1 knockdown on PTCH1 expression. Notably, miR-152 inhibitor reversed the effects of PVT1 knockdown on HSC activation. Luciferase reporter assays and pull-down assays showed a direct interaction between miR-152 and PVT1. Collectively, we demonstrate that PVT1 epigenetically down-regulates PTCH1 expression *via* competitively binding miR-152, contributing to EMT process in liver fibrosis.

## INTRODUCTION

Liver fibrosis occurs in almost all patients with chronic liver diseases (CLDs) and represents the final common pathway of virtually all types of CLDs [1]. Liver fibrosis is considered as a reversible wound-healing process and featured with the excess deposition of extracellular matrix (ECM) proteins [2]. With prolonged liver injuries in CLDs, fibrosis may progress to cirrhosis and hepatocellular carcinoma (HCC) [3]. Liver fibrosis is a major cause of morbidity and mortality worldwide, therefore, it has been a major public health concern.

The activation and transdifferentiation of hepatic stellate cells (HSCs) are pivotal events in liver fibrosis [1, 4]. To explore the effective treatments on the suppression of activated HSCs is a potential targeted therapy for liver fibrosis. Epithelial-mesenchymal transition (EMT) is a process whereby epithelial cells gradually lose their epithelial signatures, while acquiring the characteristics of mesenchymal cells. Interestingly, EMT process is involved in the activation of HSCs [5]. Activated EMT process contributes to HSC trans-differentiation and liver fibrosis *via* activating Hedgehog (Hh) signaling pathway [6]. Conversely, Hh pathway inactivation contributes to the suppression of EMT process in HSCs [7]. Patched1 (PTCH1), a member of Hh family, is a negative regulator of Hh pathway. Our previous study showed that microRNA-152 (miR-152) inhibits liver fibrosis by attenuating DNA methyltransferase 1(DNMT1)-mediated PTCH1 methylation [8].

Long non-coding RNAs (lncRNAs) are a class of transcripts (> 200 nt in length) that structurally resemble mRNAs but do not encode proteins. Emerging evidence has demonstrated that lncRNAs are frequently deregulated in various diseases and implicated in the development and metastasis of cancers [9]. The roles of lncRNAs are involved in multiple biological processes such as proliferation, apoptosis, cell migration and differentiation [9, 10]. Although many lncRNAs have been reported to be involved in the activation of HSCs [11, 12], the specific roles of lncRNAs in regulating EMT in liver fibrosis remain unclear. Recently, plasmacytoma variant translocation 1 (PVT1), a novel lncRNA, has been reported to be up-regulated in human cancers such as HCC, ovarian cancer and non-small lung cancer [13–15]. PVT1 may serve as a crucial regulator in cancers. For example, PVT1 can promote cell proliferation, cell cycling, and the acquisition of stem cell-like properties in HCC cells by stabilizing NOP2 protein [13]. Increased PVT1 level may be associated with poor prognosis in many cancers [16, 17]. Notably, PVT1 can serve as a mediator of ECM accumulation in the kidney [18], suggesting that PVT1 may be involved in fibrosis. In this study, we aimed to explore the roles of PVT1 in liver fibrosis.

## RESULTS

### Up-regulation of PVT1 in activated HSCs and liver fibrotic tissues

**Figure 1:**
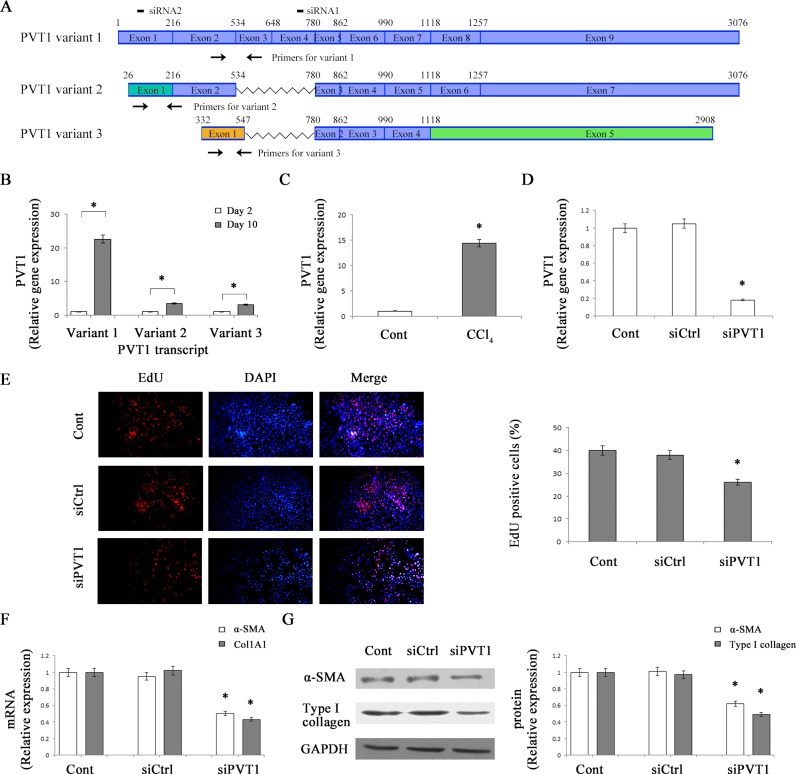
Up-regulation of PVT1 in liver fibrosis and effects of PVT1 on HSC activation *in vitro* **A.** Schematic diagram of variants of the PVT1 transcript. Color boxes indicated the exons of variants of the PVT1 transcript. Black boxes indicated the siRNAs for PVT1 transcript variant 1. Black arrows indicated the PCR primers in the variants. Notably, the sequences of the exon 1 of PVT1 transcript variant 2 were different from those in PVT1 transcript variant 1. Moreover, the sequences of the exon 1 and exon 5 of PVT1 transcript variant 3 were different from those in PVT1 transcript variant 1 and 2. **B.** PVT1 transcript variant 1, PVT1 transcript variant 2 and PVT1 transcript variant 3 expressions were detected by qRT-PCR in primary HSCs. **C.** PVT1 was analyzed by qRT-PCR in CCl_4_ mice. **D.** PVT1 was measured by qRT-PCR in primary HSCs transfected with siPVT1. **E.** Cell proliferation was determined by the EdU assay. The mRNA **F.** and protein **G.** expression levels of α-SMA and type I collagen were analyzed in primary HSCs transfected with siPVT1. GAPDH was used as internal control. *P < 0.05 compared to the control. Each value is the mean ± SD of three experiments.

It is known that isolated mouse HSCs cultured on plastic will gradually become an activated myofibroblastic phenotype during culture days [19]. To explore the biological role of PVT1 in liver fibrosis, we firstly detected the expression of PVT1 in primary 2-day-old HSCs and primary 10-day-old HSCs. As indicated by Figure 1A, there are three different PVT1 isoforms including PVT1 transcript variant 1 (NR_003368.2), PVT1 transcript variant 2 (NR_132746.1) and PVT1 transcript variant 3 (NR_132747.1) in NCBI (National Center for Biotechnology Information) database. As shown by quantitative real-time PCR (qRT-PCR) analysis, the expressions of all PVT1 variants were up-regulated at day 10 compared with those at day 2 (Figure 1B). Notably, PVT1 variant 1 is mostly up-regulated than PVT1 variant 2 and 3 in activated HSCs. Thus, PVT1 variant 1 was selected for the following analysis. Moreover, PVT1 expression was analyzed in carbon tetrachloride (CCl_4_)-induced liver fibrosis in mice. Compared with control liver, PVT1 expression in CCl_4_-treated liver was increased by 14.4-fold (Figure 1C). Our data suggest that PVT1 was increased during liver fibrosis and may play a role in liver fibrosis.

### Knockdown of PVT1 inhibits the activation of HSCs

HSC activation is characterized by enhanced cell proliferation, over-production of ECM, and de novo synthesis of α-smooth muscle actin (α-SMA) [20]. To evaluate the functions of PVT1 in liver fibrosis, primary HSCs were transfected with PVT1 siRNA (siPVT1). It was found that PVT1 level in HSCs transfected with siPVT1 was decreased by 82% relative to the control (Figure 1D). Using 5-Ethyny-2′-deoxyuridine (EdU) assays, the effects of siPVT1 on HSC proliferation were examined. PVT1 knockdown led to a significant reduction in cell proliferation (Figure 1E). Next, we examined the roles of siPVT1 on α-SMA and collagen expression. The mRNA levels of α-SMA and alpha-1(I) collagen (Col1A1) were decreased by 50% and 57%, respectively, in siPVT1 group relative to the control (Figure 1F). Consistent with mRNA data, immunoblot analysis showed that the protein levels of α-SMA and type I collagen in cells transfected with siPVT1 were reduced by 38% and 51%, respectively (Figure 1G). To further confirm the effects of PVT1 knockdown on α-SMA and type I collagen, PVT1 siRNA2 (siPVT1-2) was transfected into HSCs. As shown in Figure S1A, PVT1 expression was reduced in siPVT1-2 group. Consistent with the previous results, siPVT1-2 resulted in a significant reduction in the protein levels of α-SMA and typeIcollagen (Figure S1B). Our results suggest that PVT1 contributes to HSC activation.

### Loss of PVT1 alleviates CCl4-induced liver fibrosis

**Figure 2:**
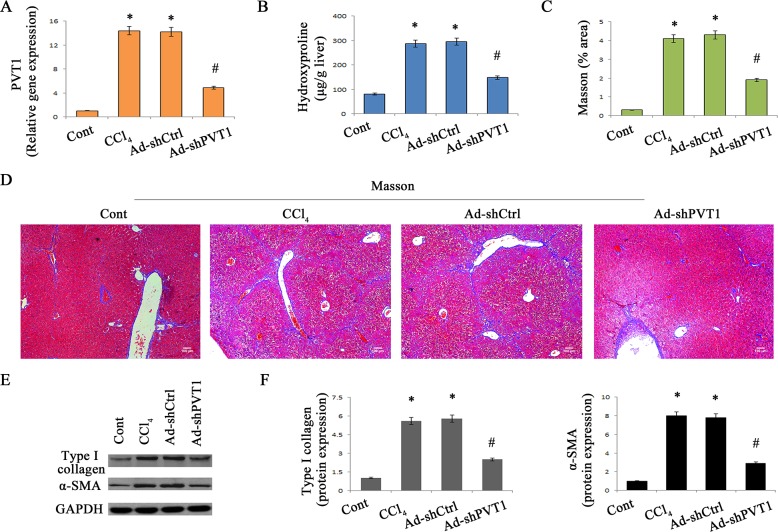
Silencing PVT1 contributes to the suppression of liver fibrosis in CCl_4_ mice **A.** Relative gene expression of PVT1 was analyzed by qRT-PCR. **B.** The level of hydroxyproline was detected in CCl_4_ mice after Ad-shPVT1 treatment. (C and D) Accumulation of collagen was assessed by Masson staining. Scale bar, 100 μm. (E and F) The protein levels of α-SMA and type I collagen were measured by Western blotting. GAPDH was used as internal control. *P < 0.05 compared to the control and ^#^*P* < 0.05 compared to the CCl_4_ group. Each value is the mean ± SD of three experiments.

To confirm the role of PVT1 in the progression of liver fibrosis *in vivo*, the effects of PVT1 knockdown on CCl_4_-induced liver fibrosis were explored. Delivery of adenoviral vectors expressing shRNA against PVT1 (Ad-shPVT1) significantly led to the suppression of PVT1 expression *in vivo* (Figure 2A). As shown by liver hydroxyproline content and Masson staining, CCl_4_-induced collagen expression was inhibited by loss of PVT1 (Figure 2B-2D). To further confirm the effects of loss of PVT1 on collagen expression, CCl_4_ mice were also treated with adenoviral vectors expressing shRNA2 against PVT1 (Ad-shPVT1-2). Notably, Ad-shPVT1-2 led to a significant reduction in hydroxyproline content in CCl_4_ mice (Figure S2). In line with these results, increased type I collagen expression caused by CCl_4_ was blocked down by PVT1 knockdown (Figure 2E and Figure 2F). Likewise, silencing PVT1 resulted in the suppression of CCl_4_-induced α-SMA expression (Figure 2E and Figure 2F).

### Silencing PVT1 attenuates liver fibrosis through suppressing EMT

**Figure 3:**
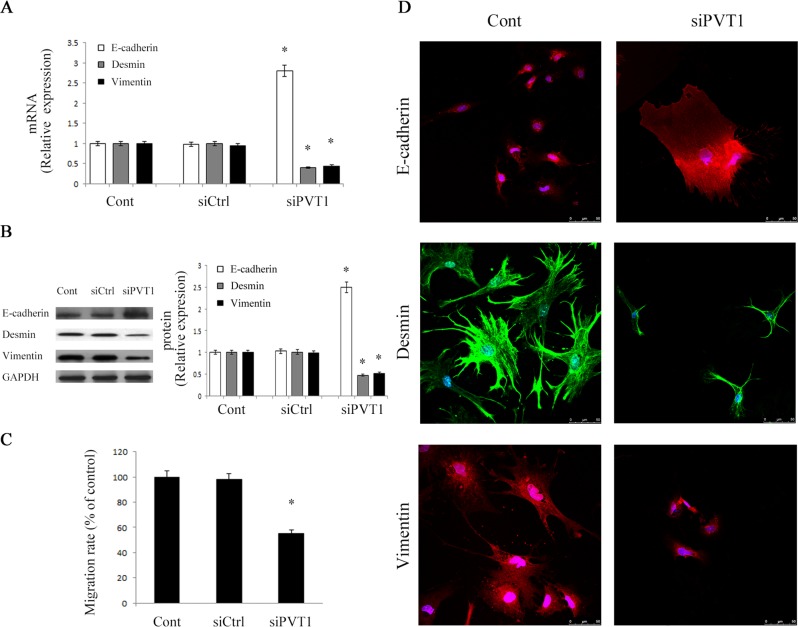
Effects of PVT1 on EMT process **A.** The mRNA levels of E-cadherin, desmin and vimentin were analyzed by qRT-PCR. **B.** The protein levels of E-cadherin, desmin and vimentin were analyzed by Western blotting. GAPDH was used as internal control. **C.** Cell migration was analyzed by transwell migration assay. Five fields of migrated cells in the lower side of transwell were counted with a microscope at ×100. **D.** Immunofluorescence staining for E-cadherin (red), desmin (green) and vimentin (red) were evaluated by confocal laser microscopy. DAPI stained nuclei blue. Scale bar, 50 μm. *P < 0.05 compared to the control. Each value is the mean ± SD of three experiments.

Next, to investigate whether EMT process was involved in the effects of PVT1, the levels of EMT markers such as E-cadherin (epithelial marker), desmin and vimentin (myofibroblastic marker) were detected in primary HSCs. The mRNA and protein expressions of E-cadherin were obviously enhanced by loss of PVT1 (Figure 3A and Figure 3B). Conversely, in comparison with control, the levels of desmin and vimentin were significantly reduced by the inhibition of PVT1 (Figure 3A and Figure 3B). Similarly, E-cadherin expression was enhanced by siPVT1-2, whereas desmin and vimentin expressions were inhibited by siPVT1-2 (Figure S1C). Then, we determined the functions of siPVT1 in cell migration. As confirmed by transwell migration assays, HSCs migration was significantly reduced by PVT1 knockdown (Figure 3C). Using immunofluorescence analysis, we further confirm the effects of siPVT1 on EMT markers. Consistent with the previous results, E-cadherin expression was increased by siPVT1, whereas the levels of desmin and vimentin were reduced in cells after siPVT1 treatment (Figure 3D). These data suggest that PVT1 promotes EMT process in liver fibrosis.

### Silencing PVT1 inhibits Hh pathway and PTCH1 methylation

**Figure 4:**
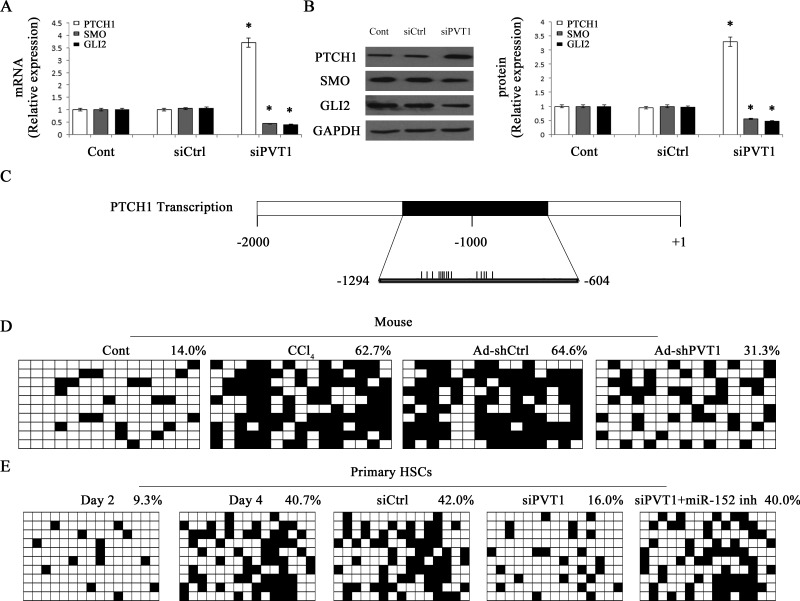
Effects of PVT1 on Hh pathway and PTCH1 promoter methylation The mRNA **A.** and protein **B.** levels of PTCH1, SMO and GLI2 were analyzed by qRT-PCR and Western blotting, respectively. **C.** A schematic representation of the promoter region amplified by bisulfide sequencing. Black box represents the region selected for bisulfite sequencing. Each vertical bar represents the presence of a CpG dinucleotide. Promoter methylation of PTCH1 was detected by bisulfide sequencing in mice **D.** and primary HSCs **E.**. Primary 2-day-old HSCs were transfected with siPVT1 for 48 h and then treated with miR-152 inhibitor for additional 48 h. The average percentage of DNA methylation was shown at the end of each row. *P < 0.05 compared to the control. Each value is the mean ± SD of three experiments.

The Hh family mainly includes Sonic Hh (SHH), PTCH1, the Smoothened (SMO) and GLI family zinc finger (GLI). To determine whether Hh pathway was involved in the effects of PVT1 on regulating EMT in liver fibrosis, Hh pathway-related genes including PTCH1, SMO and GLI2 were detected by qRT-PCR and immunoblot analysis. The expression of PTCH1 was markedly increased by siPVT1, whereas the expressions of SMO and GLI2 were reduced by siPVT1 (Figure 4A and Figure 4B). In line with these results, siPVT1-2 contributed to the increase in PTCH1 and the reduction in SMO and GLI2 (Figure S1D). All these results indicated that PVT1 knockdown led to Hh pathway inactivation. Recently, Yang *et al*. found that silencing of PTCH1 level was responsible for the activation of Hh pathway and associated with PTCH1 methylation in liver fibrosis [21]. To understand whether increased PTCH1 caused by siPVT1 was associated with DNA methylation of PTCH1 promoter, the methylation level at 15 CpG sites within the CpG island in the PTCH1 locus was analyzed by bisulfite-sequencing analysis (Figure 4C). The average rate of PTCH1 methylation was 14.0% in the control mice, whereas it was increased to 62.7% in CCl_4_-treated mice (Figure 4D). But Ad-shPVT1 treatment inhibited CCl_4_-induced PTCH1 methylation, which was just 31.3% in Ad-shPVT1 group (Figure 4D). Similar results were observed *in vitro*. PTCH1 methylation was increased in primary HSCs during culture days. The average rate of PTCH1 methylation was 9.3% at Day 2, whereas it was increased to 40.7% at Day 4 (Figure 4E), indicating that PTCH1 methylation was increasing during HSC activation. Notably, PTCH1 methylation rate was reduced to 16% in siPVT1 group relative to the control (Figure 4E). Taken together, PVT1 promotes EMT process and Hh pathway activation through PTCH1 methylation.

### PVT1 enhances PTCH1 methylation *via* miR-152

**Figure 5:**
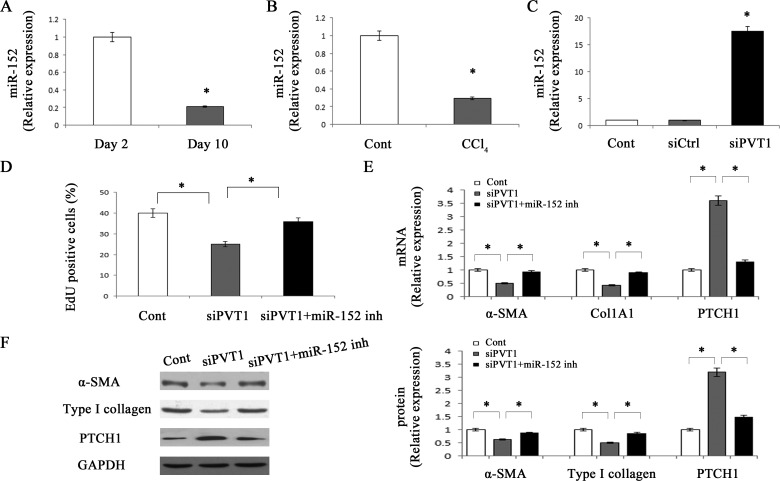
miR-152 is involved in the effects of PVT1 on HSC activation Primary 2-day-old HSCs were transfected with siPVT1 for 48 h and then treated with miR-152 inhibitor for additional 48 h. The level of miR-152 was detected by qRT-PCR in primary HSCs **A.** and CCl_4_ mice **B.**. **C.** The level of miR-152 was measured by qRT-PCR in primary HSCs transfected with siPVT1. **D.** Cell proliferation was determined by the EdU assay. The mRNA **E.** and protein **F.** expression levels of α-SMA, type I collagen and PTCH1 were analyzed by qRT-PCR and Western blotting, respectively. GAPDH was used as internal control. *P < 0.05 compared to the control. Each value is the mean ± SD of three experiments.

**Figure 6:**
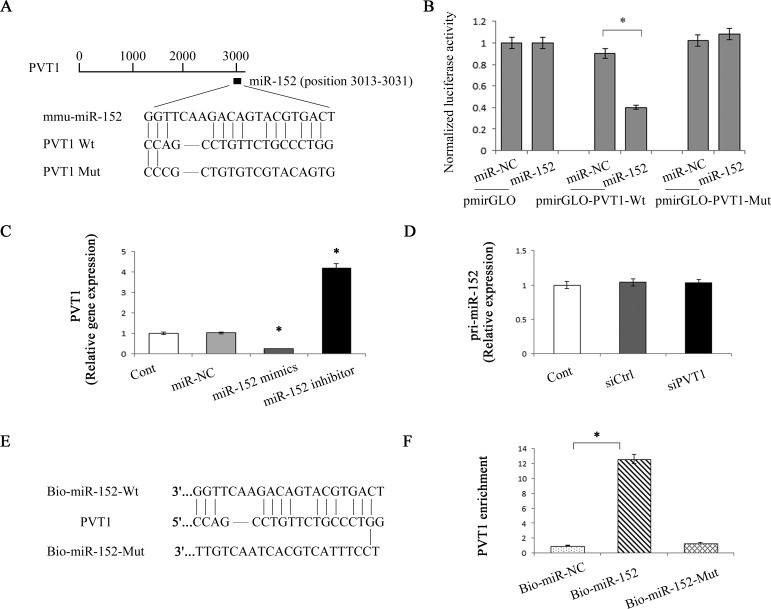
Interaction between PVT1 and miR-152 **A.** Schematic diagram of the miR-152 binding site in the PVT1 based on RNA22 software. **B.** Relative luciferase activities of luciferase reporters harboring the wild-type or mutant PVT1 were analyzed 48 h following transfection with the miR-152 mimics or miR-NC. **C.** Relative gene expression of PVT1 was analyzed by qRT-PCR in cells transfected with miR-152 mimics or inhibitor. **D.** Relative gene expression of pri-miR-152 was analyzed by qRT-PCR in cells transfected with siPVT1. **E.** Schematic diagram of wild type and the mutated form of miR-152 sequence. **F.** Pull down assay to validate the direct interaction between PVT1 and miR-152. Bio-miR-NC is not complementary to PVT1. *P < 0.05 compared to the control. Each value is the mean ± SD of three experiments.

Previously, we demonstrated that DNMT1 is a direct target of miR-152 and miR-152 contributes to the hypomethylation of PTCH1 *via* inhibiting DNMT1 [8]. Based on these, we next investigate whether miR-152 was involved in siPVT1-induced PTCH1. We firstly detected miR-152 level in primary HSCs during culture days. miR-152 level was decreased by 79% at day 10 relative to day 2 (Figure 5A). Similarly, miR-152 level was reduced in CCl_4_ group (Figure 5B). miR-152 level may be negatively correlated with PVT1 expression in liver fibrosis. The correlation between miR-152 and PVT1 was further determined in cells transfected with siPVT1. Loss of PVT1 resulted in the elevation of miR-152 level (Figure 5C). Further studies were performed to examine the role of miR-152 in the effect of siPVT1 on HSC activation. Interestingly, miR-152 inhibitor reversed siPVT1-suppressed HSC proliferation (Figure 5D). Likewise, reduced α-SMA and collagen caused by siPVT1 were inhibited by miR-152 inhibitor (Figure 5E and Figure 5F). Notably, siPVT1-induced PTCH1 was blocked down by miR-152 inhibitor (Figure 5E and Figure 5F). Consistent with these, the hypermethylation of PTCH1 was restored by miR-152 inhibitor in siPVT1-transfected cells (Figure 4E). Our data suggest that PVT1 may modulate PTCH1 methylation *via* miR-152. Bioinformatic analysis (RNA22) shows that PVT1 contains one target site for miR-152 (Figure 6A), indicating that there may be an interaction between miR-152 and PVT1. Then, pmirGLO construct was used to generate a PVT1 luciferase reporter containing the miR-152-binding sites (pmirGLO-PVT1-Wt) or mutated sites (pmirGLO-PVT1-Mut) (Figure 6A). Our results demonstrated that miR-152 mimics led to the reduction of luciferase activity of pmirGLO-PVT1-Wt in primary HSCs without affecting that of pmirGLO-PVT1-Mut (Figure 6B). The results suggest that PVT1 is a target of miR-152. In addition, miR-152 mimics induced a reduction in PVT1 expression, whereas miR-152 inhibitor caused an increase in PVT1 expression (Figure 6C). Interestingly, pri-miR-152 level was not affected by siPVT1 (Figure 6D), suggesting that PVT1 regulates miR-152 at the post-transcriptional level. To further determine the direct interaction between miR-152 and PVT1, biotinylated miR-152 (Bio-miR-152) pull-down assay was performed to confirm whether miR-152 could pull down PVT1. As shown in Figure 6E and Figure 6F, Bio-miR-152-Wt pulled down PVT1 while Bio-miR-152-Mut had no effect on PVT1, indicating a direct interaction between miR-152 and PVT1. Collectively, PVT1 inhibits PTCH1 expression *via* competitively binding miR-152.

## DISCUSSION

In this study, we found that PVT1 was increased in fibrotic liver tissues and in activated HSCs. Silencing PVT1 not only inhibited liver fibrosis *in vivo* but also caused the suppression of activated HSCs *in vitro*. Due to the loss of PVT1, Hh pathway was inactivated and EMT process was inhibited. Our results suggest that PVT1 plays a pro-fibrotic role in liver fibrosis and this is a first report. We also demonstrate that epigenetic modification of PTCH1 mediated by miR-152 is involved in the effects of PVT1 on HSC activation.

In past decades, lncRNAs have been considered as simply transcriptional “noise” or cloning artifacts. In fact, recent studies have identified large numbers of lncRNAs playing important regulatory roles that were previously reserved for proteins [22]. Recently, emerging evidences have demonstrated that lncRNAs act as important protagonists of cellular functions *via* diverse molecular mechanisms including chromatin modification, transcriptional regulation and post-transcriptional regulation [10, 11, 22]. Among post-transcriptional regulation, lncRNAs act as competing endogenous RNAs (ceRNAs) to sponge miRNAs, consequently modulating the de-repression of miRNA targets [23–27]. For example, lncRNA-growth arrest-specific transcript 5 (lncRNA-GAS5) has been shown to increase p27 protein by functioning as a ceRNA for miR-222 in liver fibrosis, resulting in the suppression of liver fibrosis [26]. Recent studies have reported that PVT1 functions as an oncogene and accelerates the progression of cancers [13, 28]. Wang *et al*. showed that PVT1 expression is distributed in both nucleus and cytoplasm [13], indicating that PVT1 may serve as a ceRNA for miRNAs. In normal breast, Paci *et al*. identified PVT1 as a ceRNA for miR-200 family [29]. In this study, whether PVT1 can function as a ceRNA for miRNAs in liver fibrosis was explored. Our results showed that PVT1 enhanced PTCH1 methylation and caused a reduction in PTCH1 expression *via* competitively binding miR-152. As confirmed by luciferase reporter assays and pull-down assays, there is a direct interaction between miR-152 and PVT1. In addition, PVT1 inhibits miR-152 in a post-transcriptional manner. Notably, all the effects of siPVT1 on HSC activation can be blocked down by miR-152 inhibitor. All the data above suggest that PVT1 can function as a ceRNA for miR-152.

DNA methylation is a type of epigenetic modifications in mammals and its correlation to pathogenesis of liver fibrosis has been well established [30, 31]. Mammalian DNA is dominantly methylated in the C-5-position of complimentary CpG bp DNMTs, including DNMT1, DNMT3a and DNMT3b [32]. DNA methylation contributes to the loss of PTCH1 in tumors [33]. Yang *et al*. reported that decreased PTCH1 expression in liver fibrosis is associated with PTCH1 hypermethylation [21]. In our study, PTCH1 hypermethylation in fibrotic liver tissues and activated HSCs was inhibited by PVT1 knockdown. Owing to the loss of PTCH1 methylation caused by PVT1 knockdown, the level of PTCH1 was restored, leading to the inactivation of Hh pathway and the inhibition of EMT process. Interestingly, siPVT1-induced PTCH1 demethylation was inhibited by miR-152 inhibitor. Our results indicated that miR-152 was responsible for the effects of PVT1 on PTCH1 methylation, which was consistent with our previous study [8]. With the restoration of PTCH1 methylation, miR-152 inhibitor reversed siPVT1-inhibited HSC activation. Taken together, PTCH1 expression is associated with its promoter methylation level and PVT1 contributes to HSC activation through regulation of miR-152 and PTCH1 methylation.

**Figure 7:**
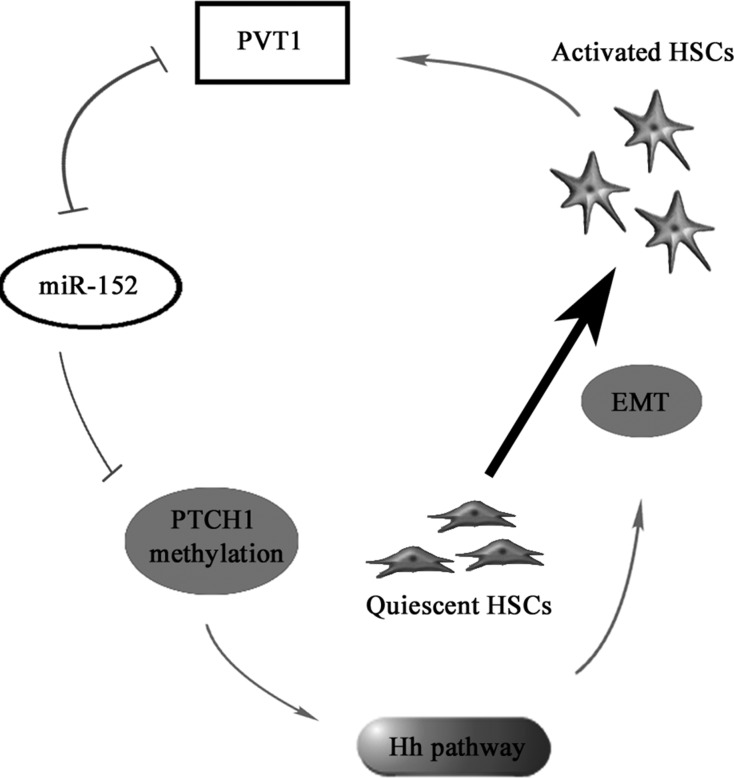
The signaling pathway was discovered in activated HSCs PVT1 induces miR-152 down-regulation, PTCH1 methylation and activation of Hh pathway, then promotes EMT process, which contributes to the activation of HSCs.

In conclusion, we demonstrate that PVT1 can epigenetically inhibit PTCH1 *via* competitively binding miR-152, contributing to activation of Hh pathway and EMT process in liver fibrosis. We also identify PVT1/miR-152/PTCH1 as a new signaling network in liver fibrosis (Figure 7).

## MATERIALS AND METHODS

### Cells

Primary HSCs were isolated as described previously [34]. Cells were cultured in Dulbecco's modified Eagle's medium supplemented with 10% fetal bovine serum, 100 U/ml penicillin and 100 U/ml streptomycin. The purity of cultures was confirmed by immunocytochemical staining for α-SMA and the purity reached > 98%. The harvested primary HSCs were studied at day 2 after isolation throughout all the studies. Primary 2-day-old HSCs were trans­fected with PVT1 siRNA or the scrambled siRNA (siCtrl) using Lipofectamine RNAiMAX (Life Technologies Co, Ltd., Grand Island, NY, USA, cat# 13778075) for 48 h in all experiments.

### CCl_4_ liver injury model

Eight-week-old male C57BL/6J mice (*n* = 6) received intraperitoneal injection of 7 μL/g of 10% CCl_4_ (Sigma-Aldrich, St. Louis, MO, USA, cat# 289116) in olive oil two times weekly for six weeks. Meanwhile, mice (*n* = 6) treated with olive oil treatment were considered as the control mice. As well as oil treatment and CCl_4_ treatment, mice additionally received CCl_4_ in combination with adenoviral vectors expressing the scrambled shRNA (Ad-shCtrl) (*n* = 6) and CCl_4_ in combination with Ad-shPVT1 (*n* = 6) or Ad-shPVT1-2 (*n* = 6). Ad-shPVT1 or Ad-shPVT1-2 (1×10^9^ pfu/100 μL) was injected every two weeks by way of the tail vein for 6 weeks. Ad-shPVT1, Ad-shPVT1-2 and Ad-shCtrl were purchased from GenePharma biotechnology (Shanghai, China). The animals were provided by the Experimental Animal Center of Wenzhou Medical University. The animal experimental protocol was approved by the University Animal Care and Use Committee. Mice were sacrificed under anesthesia at the end of six weeks and the livers were removed for further analysis. The liver tissues were used for Masson staining by fixation with 10% formalin. Quantitative analysis for the Masson-positive area was calculated from five fields for each liver slice.

### Hepatic hydroxyproline content

Liver tissues (50 mg) were homogenized in HCl and hydrolyzed at 120°C overnight. After lysate centrifugation at 12,000 g for 10 min at 4°C, the supernatant was evaporated to dryness under vacuum. The hepatic hydroxyproline content was assessed using the Hydroxyproline Colorimetric Assay kit (BioVision, San Francisco, CA, cat# K555-100). Data were normalized to liver weight.

### RNAi assay and transfection analysis

RNA interference experiments were performed following the manufacturer's protocols. siPVT1 (sense, 5′-AAGGAAGCUCUUCUUGAGC-3′; antisense, 5′-GCUC AAGAAGAGCUUCCUU-3′), siPVT1-2 (sense, 5′-GGAAUGCUAAGUUCGUAGC UU-3′; antisense, 5′-AAGCUACGAACUUAGCAUUCC-3′) and scrambled siRNA (negative control) were designed and synthesized by GenePharma. miR-152 mimics and miR-152 inhibitor were additionally synthesized by GenePharma. Moreover, siCtrl and miR-NC were used as the negative control. HSCs were transfected with siPVT1, siPVT1-2, miR-152 mimics or miR-152 inhibitor using Lipofectamine RNAiMAX at a final concentration of 10 nM.

### Immunofluorescence microscopy

Immunofluorescent staining was performed as previously described [8]. Briefly, at 48 h after primary 2-day-old HSCs were transfected with siPVT1, cells were fixed in an acetic acid: ethanol (1:3) solution for 5 min at −20°C and washed with PBS. Nonspecific binding was blocked with 5% goat serum/PBS for 1 h at room temperature, and these cells were then incubated with primary antibodies against E-cadherin (Abcam, Cambridge, MA, USA, cat# ab76055), desmin (Abcam, cat# ab15200) or vimentin (Abcam, cat# ab8978) in a humidified chamber. After washing twice in PBS, the cells were incubated with fluorescein-labelled secondary antibody (1:50 dilution; Dianova, Hamburg, Germany) in antibody dilution solution for 1 h at room temperature in the dark. The nuclei were stained with 4,6-diamidino-2-phenylindole (DAPI) in the dark for 30 min at room temperature. The slides were washed twice with PBS, covered with DABCO (Sigma-Aldrich), and examined by confocal laser scanning microscopy (Olympus, Tokyo, Japan) at 488 and 568 nm.

### Transwell migration assay

Cells were placed in the top chamber of transwell migration chambers (8 μm; Millipore, Billerica, MA, USA). After 48 h, cells which had not migrated to the lower chamber were removed from the upper surface of the transwell membrane with a cotton swab. Migrating cells on the lower membrane surface were fixed, stained, photographed and counted using a microscope at ×100. Experiments were assayed in triplicate, and ≥5 fields were counted in each experiment.

### qRT-PCR

Total RNA was extracted from cells and liver tissues using the miRNeasy Mini Kit (Qiagen, Valencia, CA, USA, cat# 217004). 50 ng of total RNA was reverse-transcribed to cDNA using the ReverTra Ace qPCR RT Kit (Toyobo, Osaka, Japan, cat# FSQ-101) in accordance with the manufacturer's instructions. Gene expression was measured by real-time PCR using cDNA, SYBR Green real-time PCR Master Mix (Toyobo, Osaka, Japan, cat# QPK-101). The PVT1 primers for PCR were listed in Table S1. The primers of Col1A1, α-SMA, E-cadherin, vimentin, desmin, PTCH1, SMO, GLI2, U6 and GAPDH were designed as described previously [8]. To detect miR-152 and pri-miR-152 expressions, the RT reactions were performed using the TaqMan MicroRNA Assays (Applied Biosystems, Foster City, CA) according to the manufacturer's instructions. The GAPDH level was used to normalize the relative abundance of PVT1 and mRNAs. U6 snRNA was used to normalize the relative abundance of miRNAs. The expression levels (2^−ΔΔCt^) of PVT1, mRNAs and miRNAs were calculated as described previously [35].

### Western blot analysis

Equal amounts of protein were subjected to sodium dodecy1 sulphate-polyacrylamide gel electrophoresis and then transferred onto membranes. The membrane was incubated with primary antibodies overnight at 4°C, then secondary IRdye800-conjugated goat anti-mouse IgG or goat anti-rabbit IgG at 37°C for 1 h. The antibodies are listed in Table S2. Antibody binding was detected using an Odyssey infrared scanner (Li-Cor Biosciences Inc., Lincoln, NE, USA).

### Methylation analysis

PTCH1 CpG island was searched in UCSC Genome Browser. About 0.5 μg genomic DNA was treated with sodium bisulfite and subjected to PCR. The PTCH1 primers for PCR were 5′-CTGGGAATTCAAGCCGGACC-3′ and 5′-TCTTTCGCTACCGGGAC CT-3′. The bisulfite-sequencing analysis was carried out as described previously [36].

### Proliferation assay

After transfection with siPVT1 and miR-152 inhibitor, HSCs were labelled with EdU for 12 h. HSC proliferative rate was detected using a Cell-Light™ EdU *In Vitro* Imaging Detection Kit (Guangzhou RiboBio Co., Ltd., cat# C10310-1) according to the manufacturer's instructions.

### Luciferase activity assay

Oligonucleotides containing target sequences of PVT1 were amplified and cloned into pmirGLO plasmids (Promega, Madison, WI, USA). PVT1 for miR-152 forward, 5′- TGCTGTTACCTGTATGCC-3′ and reverse, 5′-GCTTCATTACTTAATAAAGC-3′. To obtain the mutated constructs, the fragments harboring the mutated target region were synthesized by GenePharma. Empty plasmid pmirGLO was regarded as a negative control. Luciferase reporter plasmids plus miR-152 mimics or miR-NC were co-transfected into 293T using Lipofectamine RNAiMAX. Forty-eight hours after transfection, relative luciferase activity was examined in a luminometer using a Dual-Luciferase Reporter Assay System (Promega).

Pull-down assay with Bio-miR-152 Biotin pull-down was performed as previously described [26, 37]. Briefly, after 48 h of HSCs transfected with Bio-miR-152-wt, Bio-miR-152-mut, or Bio-miR-NC, the cells were washed with PBS followed by incubation in a lysis buffer for 10 min. To exclude RNA and protein complexes, the beads were blocked in lysis buffer including RNase-free bovine serum albumin and yeast tRNA (Sigma). After the lysates were incubated with streptavidin-coated magnetic beads (Life Technologies) at 4°C for 4 h, they were washed twice with lysis buffer, three times with the low salt buffer, and once with the high salt buffer. The bound RNAs were isolated using TRIzol reagent (Life Technologies). PVT1 expression was determined by qRT-PCR.

### Statistical analysis

Data from at least three independent experiments were expressed as the mean ± SD. Differences between multiple groups were evaluated using one-way analysis of variance. Differences between two groups were compared using a Student's *t*-test. *P* < 0.05 was considered significant. All statistical analyses were performed with SPSS software (version 13; SPSS, Chicago, IL).

## SUPPLEMENTARY MATERIALS FIGURES AND TABLES


