# Supplementary material for: Long non-coding RNA PVT1 activates hepatic stellate cells through competitively binding microRNA-152
Source: Oncotarget. 2016 Aug 30;7(39):62886–97. doi: 10.18632/oncotarget.11709 (PMC5325334; doi:10.18632/oncotarget.11709)
Supplement: Supplementary file 1 [file oncotarget-07-62886-s001.pdf]

Long non-coding RNA PVT1 activates hepatic stellate cells through competitively binding microRNA-152

Supporting Material

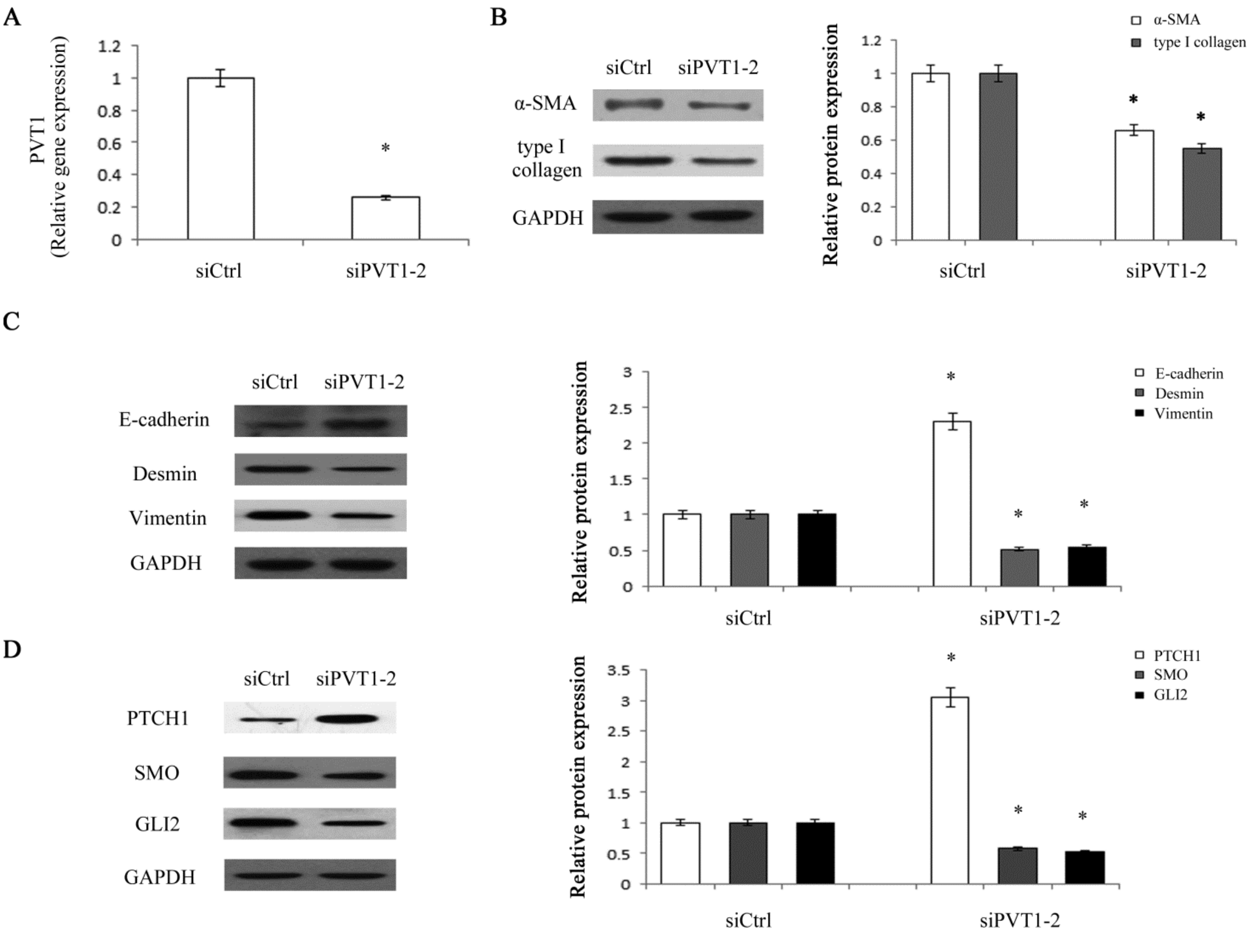

Fig.S1 Effects of siPVT1-2 on HSC activation, EMT process and Hh pathway. (A) PVT1 expression was detected by qRT-PCR in primary HSCs transfected with siPVT1-2. (B) The protein expression levels of α-SMA and type I collagen were analyzed in primary HSCs transfected with siPVT1-2. (C) The protein levels of E-cadherin, desmin and vimentin were analyzed were analyzed in primary HSCs transfected with siPVT1-2. (D) The protein levels of PTCH1, SMO and GLI2 were analyzed were analyzed in primary HSCs transfected with

siPVT1-2. GAPDH was used as internal control. *P<0.05 compared to the control. Each value is the mean ± SD of three experiments.

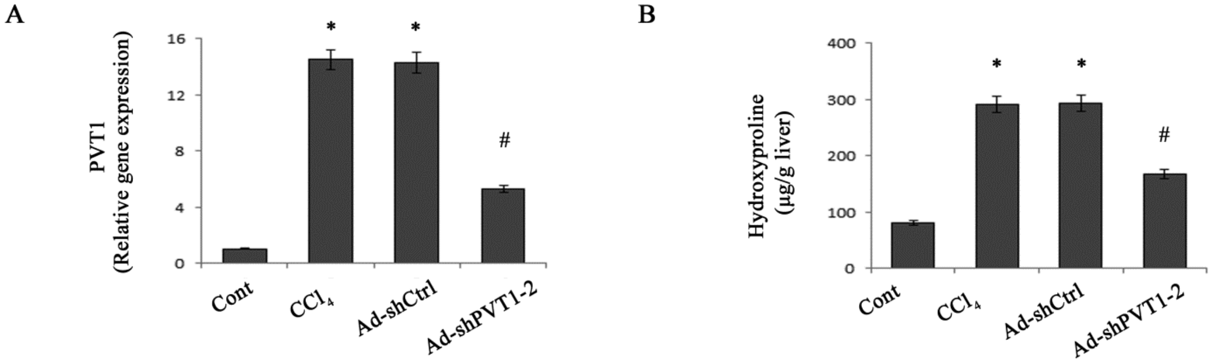

Fig.S2 Silencing PVT1 by Ad-shPVT1-2 inhibits collagen expression in CCl_4 mice. (A) Relative gene expression of PVT1 was analyzed by qRT-PCR. (B) The level of hydroxyproline was detected in CCl_4 mice after Ad-shPVT1-2 treatment. *P<0.05 compared to the control and #P<0.05 compared to the CCl_4 group. Each value is the mean ± SD of three experiments.

Table.S1 Primer sequences

Gene	Forward sequence	Reverse sequence
PVT1 transcript variant 1	5'-CCTGGATGCCCACTGAAAAC-3'	5'-GATAGACTGCTTGCCAGGGG-3'
PVT1 transcript variant 2	5'-GAGCTCCAAGTGGGACTTCT-3'	5'-GGAGGGCATCTTCTTACCGT-3'
PVT1 transcript variant 3	5'-CATGCCGGAGGCAATCCTAT-3'	5'-AAAACCACACTGGAGAGCCG-3'

Table.S2 Antibodies used in Western blotting

Antigens	Poly/monoclonal	manufacturer	Dilution
GAPDH	Monoclonal	Abcam,Cambridge, MA (ab8245)	1:2000
Type I collagen	Monoclonal	Abcam,Cambridge, MA (ab6308)	1:1000
α-SMA	Monoclonal	Abcam,Cambridge, MA (ab7817)	1:1000
E-cadherin	Monoclonal	Abcam,Cambridge, MA (ab76055)	1:1500
Desmin	Polyclonal	Abcam,Cambridge, MA (ab15200)	1:1000
Vimentin	Monoclonal	Abcam,Cambridge, MA (ab8978)	1:1500
PTCH1	Polyclonal	Abcam,Cambridge, MA (ab53715)	1:1500
SMO	Polyclonal	Abcam,Cambridge, MA (ab38686)	1:1500
GLI2	Polyclonal	Abcam,Cambridge, MA (ab26056)	1:1500
IRdye800-conjugated rabbit anti-mouse IgG	Polyclonal	Rockland, Limerick (610-431-002)	1:2000
IRdye800-conjugated goat anti-rabbit IgG	Polyclonal	Rockland, Limerick (611-131-002)	1:2000
